# First full intracorporeal robotic cystectomy and neobladder in a renal transplant recipient

**DOI:** 10.1093/jscr/rjaf009

**Published:** 2025-01-20

**Authors:** Xavier Tillou, Lisa Le Bloa, Vanja Courteille, Clemence Bechade, Thibaut Waeckel

**Affiliations:** UNICAEN, Urology and Transplantation Department, Normandie University, CHU de Caen, Avenue de la Côte de Nacre, Caen 14000, France; UNICAEN, Urology and Transplantation Department, Normandie University, CHU de Caen, Avenue de la Côte de Nacre, Caen 14000, France; UNICAEN, Anesthesiology and Critical Care Department, Normandie University, CHU de Caen, Avenue de la Côte de Nacre, Caen 14000, France; UNICAEN, Nephrology Department, Normandie University, CHU de Caen, Avenue de la Côte de Nacre, Caen 14000, France; UNICAEN, Urology and Transplantation Department, Normandie University, CHU de Caen, Avenue de la Côte de Nacre, Caen 14000, France

**Keywords:** robotic surgery, urothelial carcinoma, cystectomy, urinary diversion, renal transplantation

## Abstract

The literature regarding robotic-assisted radical cystectomy in kidney transplant recipients is limited. We present the first reported case of robotic-assisted radical cystectomy with a full intracorporeal orthotopic neobladder in a kidney transplant recipient. A 36-year-old man was diagnosed with muscle-invasive urothelial carcinoma 12 years after kidney transplantation. His immunosuppressive regimen consisted of everolimus, mycophenolate mofetil, and prednisolone. After cystectomy and left lymph node dissection, we used a U-shaped neobladder technique slightly modified to adapt to the fixed position of the renal transplant ureter. The surgical time was 305 min, and the blood loss was 200 ml. The patient was discharged 16 days after hospitalization with no surgical complications. Histological analysis revealed no UC (pT0N0) with disseminated carcinoma *in situ*. Seven months after the surgery, no signs of recurrence or distant/lymph node metastasis were observed. No urinary leakage with complete bladder emptying was reported. Serum creatinine clearance rate was 51 ml/min. Immunosuppressive regimen was not modified after surgery.

## Introduction

The treatment of organ-confined muscle-invasive bladder cancer (MIBC) is radical cystectomy combined with urinary diversion [1]. In kidney transplant recipients (KTR), surgery is difficult because of the presence of the graft in the pelvic space. Robot-assisted radical cystectomy (RARC) with an ileal neobladder is emerging as an alternative surgical strategy to open surgery [2]. RARC has been shown to reduce perioperative blood loss, shorten hospital stays, and decrease the use of analgesia [3]. However, reports of RARC in KTR are scarce. Here, we present the first reported case of RARC with a full intracorporeal orthotopic neobladder (ON) in a KTR.

## Case presentation

**Figure 1:**
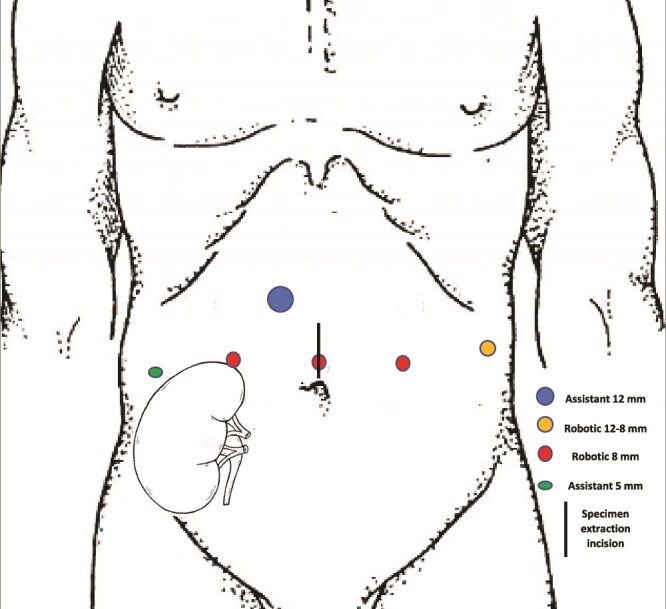
Adapted port placement for robot assisted procedure.

A 27-year-old man received a brain-dead donor left kidney transplant in the right iliac fossa after three years of hemodialysis due to malignant arterial hypertension in a single-born kidney. The immunosuppressive regimen consisted of tacrolimus, mycophenolate mofetil, and prednisolone with induction by thymoglobulin. In the following years, his serum creatinine level was 80–150 μmol/L. Tacrolimus was switched to Everolimus because of a BK virus infection with no recurrence. He presented with gross hematuria 12 years after the renal transplantation (RT) with no professional exposure to chemicals. Tobacco consumption was stopped three years before RT (20 cigarettes/day for 13 years). Cystoscopy revealed a 15 mm papillary tumor of the bladder with multiple smaller lesions. The urine cytology was compatible with high-grade urothelial carcinoma (UC). Thoracoabdominal CT scan and MRI did not reveal any lymph nodes or distant metastases. A complete transurethral resection was performed. Histological analysis revealed high-grade invasive UC with carcinoma *in situ* (CIS). The patient decided to undergo RARC with ON because of his young age and refusal of a urostomy. Before the surgery, the patient was taught clean intermittent self-catheterization and regular bicarbonate intake. Surgery was performed using the da Vinci X Surgical System via a transperitoneal approach. The patient was 167 cm tall and weighed 58 kg. He was treated preoperatively with amoxicillin-clavulanic acid (3 g/day) for urinary *Staphylococcus aureus* infection. He was placed in a 25° Trendelenburg position. Robotic ports were placed as shown in Fig. 1. First, the left ureter was dissected on the right side of the bladder, cut between two Hem-o-lok clips, and the edge was sent for frozen section diagnosis as well as the edge of the urethral section. The transplant ureter was found attached to the pubic bone and clipped with a Hem-o-lok. The bladder and prostate were removed as single specimens and were placed in an endobag. A left extended iliac lymph node dissection was performed. We used a U-shaped neobladder technique as previously described [4], slightly modified to adapt to the fixed position of the renal transplant ureter. The ureter was sutured to the neobladder before closing the right side of the anterior wall on a mono-J ureteral catheter passed through the closing of the right anterior plates and through the 5 mm assistant trocard. The neobladder was filled with 100 ml saline to confirm waterproofing. A passive drainage tube was placed in the pelvic area through a 12-8 mm robotic port. The surgical time was 305 min, and blood loss was 200 ml. The patient received oral and intravenous immunosuppressive drugs on the day of surgery and intravenous dexamethasone (8 mg). From post-surgery days (PSD) 1–3, the drugs were administered intravenously, except everolimus, because no intravenous form was available. To address this withdrawal, the corticosteroid dose was increased to 10 mg/day. The gastric tube was removed, and the original immunosuppressive regimen was resumed on PSD 7. The patient's urine output, mainly excreted through the single-J stent, remained stable throughout, at over 2000 ml/day, while the patient's serum creatinine level fluctuated between 106–176 μmol/L during the hospital stay. The drainage tube was removed on PSD 10. The ureteral single-J stent was removed 14 days after surgery. The patient was discharged 16 days after hospitalization. A clean intermittent catheterization procedure was initiated on PSD 30 to prevent metabolic acidosis after bladder catheter removal. Histological analysis of the resected specimens revealed no UC (pT0N0) with disseminated CIS. Seven months after surgery, no signs of recurrence or distant/lymph node metastasis were observed. No urinary leakage was reported with complete bladder emptying with no clean intermittent catheterization. The serum creatinine clearance rate was 51 ml/min.

## Discussion

The prevalence of UC is higher in solid organ recipients, including KTRs [5]. The management of MIBC in KTR is challenging for several reasons: choice of the surgical approach according to place taken by the renal transplant, choice of the urinary diversion depending on the anatomy of the renal transplant ureter, potential metabolic drawbacks of a neobladder in a patient with potentially chronic renal insufficiency, and management of the immunosuppressive regimen after surgery.

The choice of the surgical approach depends on a careful abdominal examination with renal graft palpation to evaluate the feasibility of the chosen surgical technique. RT surgery reports must be carefully reviewed to understand the position of the renal graft ureter. The laparoscopic approach has been proven to decrease evisceration, whereas immunosuppressive treatment with mTOR inhibitors has been suspected of compromising surgical wound healing and favoring evisceration [6]. However, this was not confirmed by Cooper in a pooled analysis of three randomized controlled trials [7]. Based on the literature, there is no evidence of any case report exploring the feasibility of radical cystectomy and full intracorporeal urinary diversion in KTR. We have not experienced surgical complications, but Ishiyama, in their case report of robotic cystectomy and extracorporeal neobladder, reported urinary leakage of the ureteroneobladder anastomosis with a subsequent abscess formation leading to a redo surgery [8]. To our knowledge, this is the first report of intracorporeal neobladder construction in a patient who underwent RARC and was a KTR. The long-term outcomes of ON in the present case must be confirmed. However, the risk of developing metabolic acidosis from the resorption of excreted metabolites and the original chronic renal disease state must be considered when attempting to apply this method.

## Data Availability

The data that support the findings of this study are available from the corresponding author upon reasonable request.
